# The Uncontrollable Mortality Risk Hypothesis: Theoretical foundations and implications for public health

**DOI:** 10.1093/emph/eoae009

**Published:** 2024-05-09

**Authors:** Richard D Brown, Gillian V Pepper

**Affiliations:** Psychology Department, Northumbria University, Newcastle, UK; Psychology Department, Northumbria University, Newcastle, UK

**Keywords:** public health, risk perceptions, health behaviours, socioeconomic inequality, uncontrollable mortality risk, perceived control

## Abstract

The ‘Uncontrollable Mortality Risk Hypothesis’ employs a behavioural ecological model of human health behaviours to explain the presence of social gradients in health. It states that those who are more likely to die due to factors beyond their control should be less motivated to invest in preventative health behaviours. We outline the theoretical assumptions of the hypothesis and stress the importance of incorporating evolutionary perspectives into public health. We explain how measuring perceived uncontrollable mortality risk can contribute towards understanding socioeconomic disparities in preventative health behaviours. We emphasize the importance of addressing structural inequalities in risk exposure, and argue that public health interventions should consider the relationship between overall levels of mortality risk and health behaviours across domains. We suggest that measuring perceptions of uncontrollable mortality risk can capture the unanticipated health benefits of structural risk interventions, as well as help to assess the appropriateness of different intervention approaches.

## THE PUBLIC HEALTH ‘PUZZLE’ OF PERSISTENT HEALTH INEQUALITIES

Despite improved access to healthcare and the widespread use of health informational campaigns in economically developed countries, socioeconomic status remains associated with long-term health outcomes [1–3]. In a systematic review of 283 public health studies, Niessen *et al.* [4] found significant support for a negative association between socioeconomic status and the prevalence of non-communicable diseases. Individuals with lower socioeconomic status typically experience poorer health, higher rates of chronic diseases, and reduced life expectancy compared to those with higher socioeconomic status [5]. Notwithstanding the role that disparities in the availability of resources and information can play in establishing social gradients in health, such gradients still exist in areas where they should theoretically be mitigated by freely available healthcare and broadly disseminated health campaigns [6]. For example, the high levels of expenditure devoted to freely available healthcare in Scandinavian countries do not translate into systematically smaller inequalities in health outcomes [7]. This public health ‘puzzle’ highlights the inability of existing theory to fully account for the presence of socioeconomic gradients in health outcomes. Addressing these socioeconomic gradients is crucial for achieving fairer public health outcomes.

Many of these differential health outcomes are affected by disparities in preventative health behaviours. For example, those who occupy lower socioeconomic positions report lower rates of exercise, use of medical services, and adherence to treatment. They also have poorer diets and higher rates of smoking [8–13]. In a further review of the relationships between socioeconomic status, health behaviours and mortality rates, it was found that smoking, alcohol consumption, physical activity and diet are all significant contributors to socioeconomic gradients in health [14]. An answer to the public health puzzle of persisting socioeconomic gradients in health outcomes will therefore require an explanation of socioeconomic gradients in health behaviours.

Pampel *et al.* [3] proposed nine categories of explanation for socioeconomic gradients in health behaviours: deprivation and stress, fewer benefits of health behaviours at lower socioeconomic positions, latent traits such as attraction to risk, class distinctions, lack of knowledge, sense of agency, aids to health behaviours, community opportunities and social support and influence. Pepper and Nettle [15] subsequently categorized these into three distinct groups, inspired by Tinbergen’s four levels of explanation [16]. First, there are constraint-based explanations for not performing preventative health behaviours, which Pepper and Nettle class as non-adaptive (Glossary Term 1). In these types of explanations, a lack of preventative health behaviour results from constraints such as having insufficient means to pursue healthy behaviours or a lack of knowledge regarding health risks. The second category is that of proximate explanations (Glossary Term 2), which specify the mechanisms associated with lower rates of investment in preventative health behaviours. Traits such as attraction to risk, shorter time horizons or a reduced sense of agency may constitute psychological mechanisms that lead to a disinvestment in preventative health behaviours [17]. However, if these explanations are evoked to explain inequalities in preventative health behaviours, they are incomplete, because they do not explain why there are socioeconomic gradients in the presence of the traits themselves. Third, there are ultimate explanations (Glossary Term 3) for socioeconomic gradients in health behaviours that suggest that those in lower socioeconomic positions receive fewer benefits from investing in preventative health behaviours than their more affluent counterparts. This final category of explanation suggests that less healthy behaviour is a ‘contextually appropriate’ response to environmental cues of risk, which may be delivered via the proximate mechanisms listed above [18]. Importantly, the application of proximate and ultimate labels to explanations for health behaviour emphasizes that explanations in these categories are not mutually exclusive—though they are often treated as such [3].

We argue that, given the pervasiveness of socioeconomic gradients in preventative health behaviour, researchers must go beyond offering constraint-based or proximate accounts. We must provide ultimate explanations to identify effective strategies for improving health behaviour. To address social disparities in preventative health behaviours, we need accounts of why the psychological mechanisms that produce these behaviours may have evolved. Instead of basing interventions solely on ‘constraint-based’ explanations of health inequalities (e.g. assuming a lack of knowledge and providing educational material) or providing interventions to mitigate the presence of specific proximate mechanisms (e.g. mindfulness interventions to reduce delay discounting), an evolutionary health perspective may look to identify and address the ultimate causes of health inequalities. Such an account is provided by Nettle [19] in a behavioural ecological model (discussed below) for explaining the presence of social gradients in preventative health behaviours and provides the basis for the Uncontrollable Mortality Risk Hypothesis.

## THE UNCONTROLLABLE MORTALITY RISK HYPOTHESIS

Uncontrollable mortality risk reflects that portion of mortality risk that cannot be mitigated by an individual allocating effort to preventative health behaviour. Though people experience varying levels of mortality risk, the degree to which these risks are individually controllable is of central importance because it determines the extent to which it may be possible for behavioural efforts to help avoid potentially fatal consequences. Nettle [19] stated that the optimal (Glossary Term 4) individual investment in health behaviour should be less for people of lower socioeconomic status because they are typically exposed to higher levels of uncontrollable mortality risk. The rate of uncontrollable mortality risk within an environment is expected to determine the optimal amount of energy that is worth investing in health. A broad range of evidence supports the assumption that people of lower socioeconomic status are exposed to greater levels of health-damaging environmental risk. This can include exposure to air pollutants, inadequate housing, poor water quality, noise exposure, hazardous waste and violence and injury [20–28]. Nettle’s model assumes that there is a trade-off between allocating resources to preventative health behaviours and investing in other activities that might contribute towards one’s fitness (Glossary Term 5). Activities that could potentially compete with an individual’s behavioural investment in health might include accruing resources, increasing status or dominance, developing social bonds, searching for a mate, or any other fitness-enhancing activity. The reduced potential payoff from engaging in preventative health behaviours, in combination with the trade-offs, makes the optimal investment in health less for those exposed to uncontrollable risks, which are more common at lower socioeconomic positions. This produces a secondary increase in mortality risk due to reduced investment in preventative health behaviours. Therefore, initial inequalities in health outcomes due to differential exposure to uncontrollable risk are worsened by a reduction in preventative health behaviour, producing a compound effect, which further entrenches health poverty.

**Figure 1.**
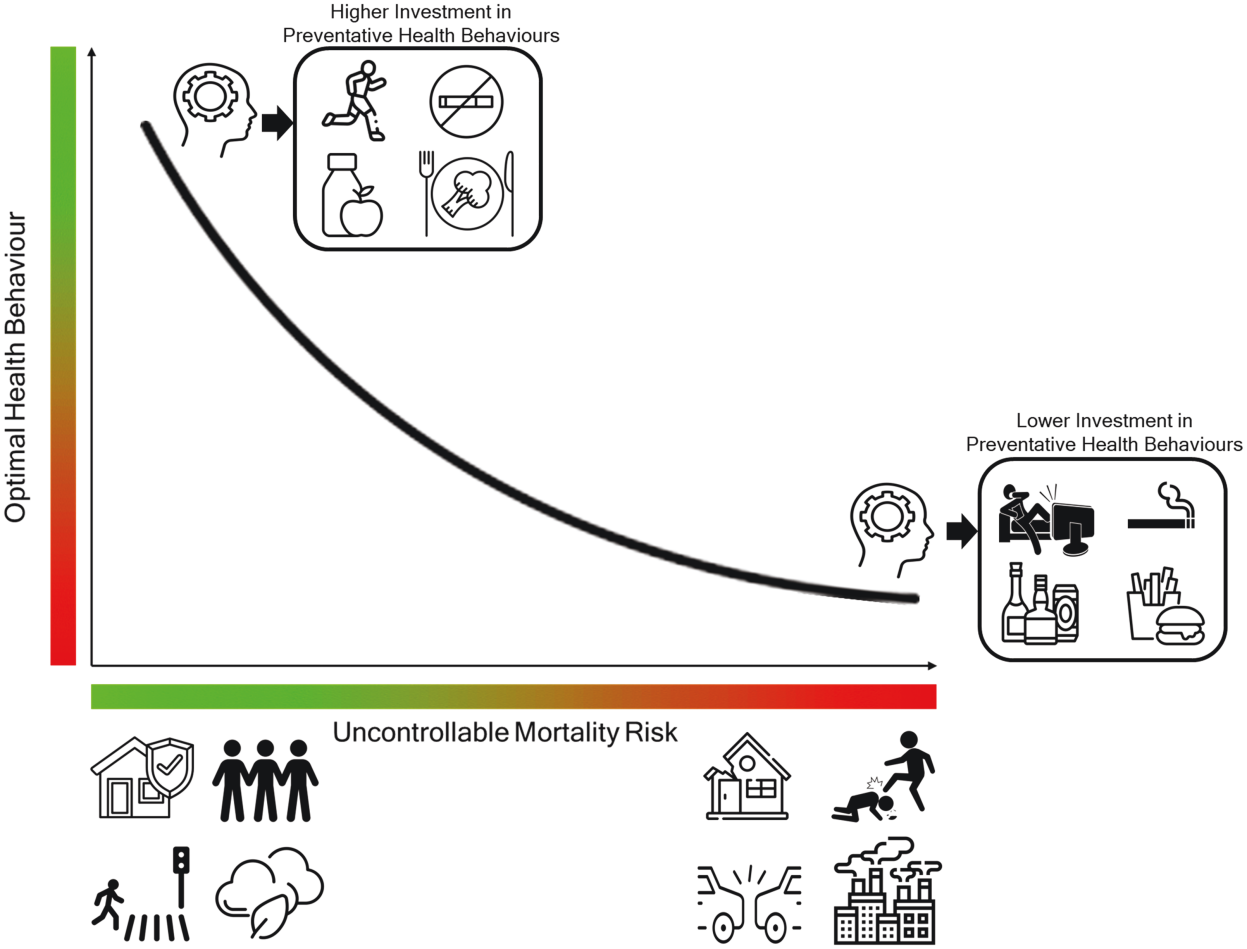
Infographic representing the Uncontrollable Mortality Risk Hypothesis of health behaviour.

Calculating objective mortality risk at the level of the individual is challenging. Objective measures of mortality risk typically operate at the aggregate level of a chosen population (e.g. area level risk of traffic fatality, or gender-based risk of violent crime) and may not provide an accurate appraisal of personal risk. Furthermore, perceptions of mortality risk often do not align with objective levels of exposure to risk. For example, a *primary bias* of risk perception has consistently been reported in which people typically overestimate rare risks to their health and underestimate common risks [29, 30]. Slovic [31] found that people often overestimate their risk of dying as a result of homicide or natural disaster, but underestimate the likelihood of dying due to diabetes, cancer or stroke. In addition, humans may not be accurately attuned to particular mortality risks that are evolutionarily novel. If a specific risk was absent or not commonly associated with death, illness or injury in our ancestral environment, the accuracy of our perceptions of this risk may be limited [32]. This suggests a possible mismatch between our perceptual responses to modern risks and the ancestral environments in which these psychological mechanisms evolved [33]. For example, we may be less adept at accurately perceiving the degree of mortality risk posed by industrial air pollution compared with interpersonal violence, as the former is a novel risk that was not directly linked to survival threats in our evolutionary history [34]. The Uncontrollable Mortality Risk Hypothesis assumes that psychological mechanisms should respond to the presence of environmental cues of risk to determine the optimal level of investment in preventative health, resulting in differences in motivation to invest in health effort. We suggest that these differences in motivation arise in response to differences in perceived levels of exposure to uncontrollable mortality risk. The oft-reported misalignment between objective and perceived levels of risk emphasizes the importance of capturing perceptions of mortality risk, and not just objective levels of exposure. Modelling this relationship between perceptions of control over risk and health behaviours provides the basis for the Uncontrollable Mortality Risk Hypothesis: that those who perceive themselves as being more likely to die due to factors beyond their control should be less motivated to invest in preventative health behaviours (see Fig. 1) [35]. It also provides the basis for a concept we call the double dividend of safety: improving people’s safety will provide the additional secondary benefit of improving their health behaviour.

### Theoretical origins of the Uncontrollable Mortality Risk Hypothesis

The Uncontrollable Mortality Risk Hypothesis draws on terminology and theoretical resources from both evolutionary biology and life history theory. Classical life history theory is based on optimization models that aim to explain variation in growth and reproduction at a species level in terms of maximizing fitness (Glossary Term 5) within an environment [36, 37]. Life history theory has been extended from the species level to the individual level to predict that an organism should respond to environmental cues and adapt their energy and resource allocation strategies accordingly [38–40]. Our specific focus on health behaviour (rather than energetic allocation to growth, reproduction or somatic maintenance) at the individual level (rather than the species level) differentiates the Uncontrollable Mortality Risk Hypothesis from the life history theory which inspired it. Furthermore, the behavioural ecological model from Nettle [19] underlying the Uncontrollable Mortality Risk Hypothesis, along with subsequent research [18, 35, 41, 42], previously referred to ‘extrinsic mortality risk’, whereas ‘uncontrollable mortality risk’ has been used more recently to refer to mortality risk that cannot be reduced by behaviour [43–46]. This is because the definition of ‘extrinsic mortality risk’ employed by evolutionary models for understanding senescence [47–51] differs from that of human health behaviour literature relevant to the Uncontrollable Mortality Risk Hypothesis [15, 18, 19, 41, 44, 46, 52–54]. The former typically defines ‘extrinsic mortality risk’ as an age and condition-independent component of environmental risk caused by external hazards such as predation, parasitism and inclement weather [19, 51]. The Uncontrollable Mortality Risk Hypothesis broadens the focus from the age, or physiological state of the organism, to the level of behavioural control that an individual has in mitigating their own risk of death. It is for this reason that we refer to the behavioural treatment of extrinsic mortality risk as ‘uncontrollable mortality risk’.

### Evidence for the Uncontrollable Mortality Risk Hypothesis

Using a novel measure of perceived uncontrollable mortality risk in an online survey of US adults, Pepper and Nettle [41] empirically tested the behavioural ecological predictions of the Uncontrollable Mortality Risk Hypothesis. They found that the positive relationship between subjective socioeconomic position and health effort (an association that is commonly reported in public health literature [1–3]) was mediated by perceived uncontrollable mortality risk. This finding is supported by our recent replication study [45], which found that perceived uncontrollable mortality risk partially mediated the positive relationship between subjective discretionary income and health effort. Our recent mini meta-analysis found that perceived uncontrollable mortality risk has been repeatedly shown to predict lower self-reported health effort [44–46], providing additional support for the assumptions of the Uncontrollable Mortality Risk Hypothesis. Levels of perceived uncontrollable mortality risk were also shown to be higher when taking the threat of COVID-19 into account [42, 55]. During the first UK lockdown in response to the COVID-19 pandemic, perceived uncontrollable mortality risk was associated with lower reported adherence to Government advice on physical activity, diet and smoking [42]. This evidence for the Uncontrollable Mortality Risk Hypothesis shows that perceptions of uncontrollable mortality risk are a consistent predictor of health effort, though we acknowledge that many other factors also influence the relationship between environmental risk and health behaviour.

Experimental evidence supporting the Uncontrollable Mortality Risk Hypothesis is provided by Pepper and Nettle [35]. The authors conducted three experiments that primed perceptions of uncontrollable mortality risk by providing participants with manipulated personalized life expectancy projections and stating whether these were or were not caused by controllable individual behaviours. They found that priming perceptions of uncontrollable mortality risk influenced a subsequent health decision—that of choosing a healthy food reward versus an unhealthy alternative. Whilst this is the only experimental research directly investigating the effects of perceived uncontrollable mortality risk on health perceptions and behaviours, research exploring the broader impact of structural changes to risk exposure offers support for the Uncontrollable Mortality Risk Hypothesis. For example, a neighbourhood renewal programme in the North East of England that made general improvements to living conditions (such as addressing damp and draughty housing) led to improved perceptions of safety and a decline in smoking [56]. Similarly, a regeneration programme involving deprived communities in Glasgow reported that improvements to internal housing conditions resulted in a lower likelihood of smoking and a higher likelihood of eating more fruit and vegetables [57]. Insufficient research has been conducted to determine which specific categories of risk exposure, when addressed, would most likely result in the greatest reductions in perceived uncontrollable mortality risk and subsequent improvements to health behaviours. However, evidence from the World Risk Poll shows that traffic accidents and crime remain the greatest perceived sources of risk in daily life globally [58]. Similarly, a systematic review of research on neighbourhood safety factors in the USA found that levels of traffic safety and crime were most relevant to health behaviours, such as physical activity among older adults [59]. These categories of risk exposure present potential targets for structural risk interventions which could lead to a reduction in perceived uncontrollable mortality risk, and improve health behaviours.

## IMPLICATIONS FOR PUBLIC HEALTH

### Highlighting the immediate benefits of healthy behaviours

The implications of the Uncontrollable Mortality Risk Hypothesis draw attention to the potential advantages of highlighting the immediate benefits of healthy behaviours. This is particularly important in circumstances where individuals may be less motivated to invest in preventive health measures due to an inaccurately held belief that they are unlikely to live long enough to enjoy the long-term benefits. In such cases, emphasizing the benefits that they are likely to experience more immediately may help to incentivise healthy behaviours. For example, instead of highlighting that a healthy diet may improve health in old age, health messages could emphasize that increased fruit and vegetable intake gives you visibly more attractive skin within 6 weeks [60]. Or they might emphasize that increased fruit and vegetable consumption has been linked to improved fertility in women and better semen quality in men [61]. Research suggests that it can be more effective to communicate the shorter-term benefits associated with quitting smoking (increasing stamina and saving money) than the longer-term benefits (healthier teeth and lungs over time) when encouraging smoking cessation [62]. By highlighting the shorter-term benefits of a healthy lifestyle, health messages may incentivise increased investment in positive health behaviours, even in those who perceive their longevity to be largely beyond their personal control. However, this raises ethical questions about whether it is right to tailor health information, with the aim of changing health behaviours, in situations where people’s health perceptions accurately reflect their degree of exposure to uncontrollable risks. As discussed above, the Uncontrollable Mortality Risk Hypothesis explains how lower investment in health behaviour can be a ‘contextually appropriate’ response to environmental cues of risk [18]. It may be considered unethical to tailor the provision of information to emphasize shorter-term benefits in order to encourage health behaviours among people who are objectively less likely to enjoy the longer-term benefits. More generally, health interventions that emphasize the consequences of lifestyle-related risk factors can encourage the perception that people *can and should* alter their behaviour, plausibly contributing to the view that people are morally responsible for their health [63]. This potential moralization of health promotion may contribute to misguided beliefs about what healthcare interventions are appropriate and draw attention away from more effective solutions, such as addressing exposures to uncontrollable risk at a structural level.

### Addressing structural disparities in risk exposure

Chater and Loewenstein [64] recently argued that researchers and policymakers across the behavioural sciences have typically sought to frame societal issues in individual (‘i-frame’), not systemic (‘s-frame’) terms. Numerous health interventions have aimed to address behaviours cheaply and effectively by influencing individual choices. For example, a large body of research has investigated the benefits of calorie labelling for tackling obesity [65–67], whilst others have argued that adopting a more systemic approach would have a greater impact, such as increasing the tax on sugar [68–70]. Given the complex challenges of researching and enacting systemic change, there is likely to be a tendency for researchers and policymakers alike to opt for an i-frame approach rather than an s-frame approach. For example, simply making people (including policymakers) aware of the option of an i-frame approach to tackling climate change (behaviourally nudging people towards green energy) has been found to reduce support for more systemic change (carbon tax) [71]. A similar distinction exists between high- versus low-agency health interventions [72]. For example, interventions that focus on delivering advice and encouragement to adopt healthier lifestyles typically require high levels of agency for individuals to engage with information and change target behaviours. In contrast, interventions such as food reformulation of high-fat-sugar-salt products, or fortifying processed foods with essential nutrients, require recipients to use little or no agency to benefit. Health interventions that require a low degree of agency for individuals to benefit are likely to be most effective and equitable. The Uncontrollable Mortality Risk Hypothesis suggests that those in lower socioeconomic positions, who are generally exposed to greater levels of uncontrollable risk, are likely to be in greater need of effective health interventions that facilitate positive health behaviours. However, the ability to exercise personal agency often relies on time, physical, and mental resources, which are usually influenced by socioeconomic factors [72]. Therefore, high-agency health interventions are likely to exacerbate existing socioeconomic gradients in health. Overemphasising the importance of i-frame and high-agency approaches to improving preventative health behaviours may inadvertently lead people to blame individuals for being unable to overcome the effects of uncontrollable features of their environment. Furthermore, fixating on how best to influence health choices may direct academic and public health attention away from addressing structural disparities in risk exposure.

### Cross-domain benefits to tackling structural disparities of risk

**Figure 2.**
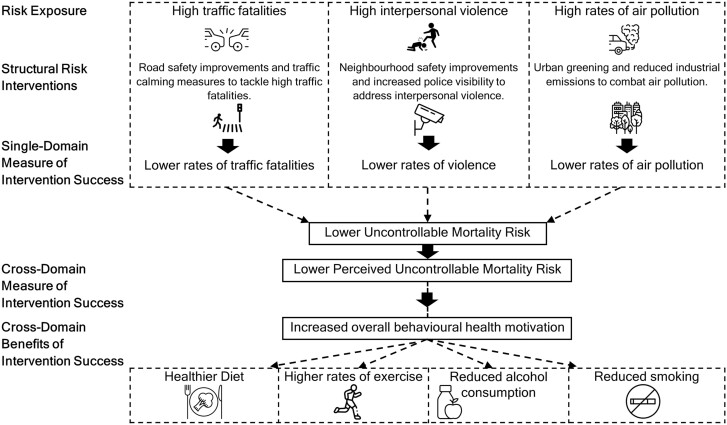
Infographic highlighting the benefits of cross-domain tracking of behavioural health changes resulting from structural risk interventions.

What might perceived risk of natural disaster or interpersonal violence have to do with motivation to eat healthily or quit smoking? The Uncontrollable Mortality Risk Hypothesis suggests that higher levels of perceived uncontrollable mortality risk *overall* should be associated with a lower *general* investment in healthy behaviour. Thus, the hypothesis extends beyond the individual relationships between specific sources of risk and the health behaviours most obviously related to those sources of risk. When assessing the impact of structural change to exposure to risk, interventions based on previous fear appeal literature (such as Protection Motivation Theory [73–76] and the Extended Parallel Process Model [77–79]), or those lacking a broader consideration of causal pathways of behaviour, are unlikely to go beyond assessing the success of an intervention in terms of the direct links between specific risks and single, directly related, health behaviours. For example, in an environment with a high number of traffic fatalities, as well as high rates of interpersonal violence and air pollution, there may be less motivation to improve healthy eating behaviour, if such a change is believed to have little impact on overall mortality risk. However, if significant efforts were made to diminish multiple sources of uncontrollable risk, this may lead to improvements in a range of health behaviours seemingly unrelated to the sources of risk. For example, eating a healthier diet may not be an immediately obvious result of improvements to road safety in one’s environment. However, an improved diet may occur because of increased health motivation resulting from a reduction in overall perceived uncontrollable mortality risk (see Fig. 2). We argue that greater attention should be given to cross-domain tracking of behavioural changes resulting from interventions. Clusters of unhealthy lifestyle behaviours are common, yet most health intervention research has addressed risk factors as categorically separate entities [80]. It has previously been suggested that more remains unknown than known about how to change multiple health behaviours both at an individual and population level [81, 82], leading to calls for greater attention to be given to the science of multiple health behaviour change [80, 83]. Measuring perceptions of uncontrollable mortality risk over time, alongside multiple health behaviours, may help to capture the effects of changes in exposure to different sources of risk (brought about by both policy changes and naturally occurring shifts in environmental risk). This may help to map out the pathways between structural changes in exposure to risk and seemingly unrelated health behaviours.

### Understanding the public’s ‘general sense’ of risk

Recent research into the drivers of perceived uncontrollable mortality risk suggests that it broadly reflects a ‘general sense’ of one’s environment that is influenced by perceived exposure to risk, as well as the level of personally available resources that someone has to avoid threats to their health and longevity [54]. Similarly, the 2023 Safety Perceptions Index (SPI) report found a rise in what it calls ‘ambiguous risk’—people’s general sense that risk exists in the world around them but cannot be precisely defined [84]. The SPI uses data from the World Risk Poll, which involves over 125,000 interviews conducted in 121 countries, to provide a comprehensive assessment of perceptions of risk across the world [84]. The 2023 SPI report suggested that this rise in perceptions of ambiguous risk may reflect a societal response to the COVID-19 pandemic [84]. However, the exact drivers of this rise in perceived ambiguous risk are unknown and could be a consequence of a changing media landscape that has an increased tendency to amplify misleading or inconclusive information [85].

Further study of what informs the public’s general sense of risk (by using measures such as perceived uncontrollable mortality risk and the SPI’s construct of ambiguous risk) may identify useful targets for interventions aimed at reducing risk exposure. For example, objective measures of risk, using data from the Global Burden of Diseases, Injuries, and Risk Factors Study (GBD) have been found to predict levels of perceived uncontrollable mortality risk, although not as strongly as one might expect, suggesting that more research is needed [44]. Identifying objective measures of risk exposure that impact the public’s general sense of risk may indicate the structural changes to risk exposure that will most effectively harness the double dividend of safety: the initial risk reduction, plus the subsequent secondary benefits from improved health behaviours.

**Figure 3.**
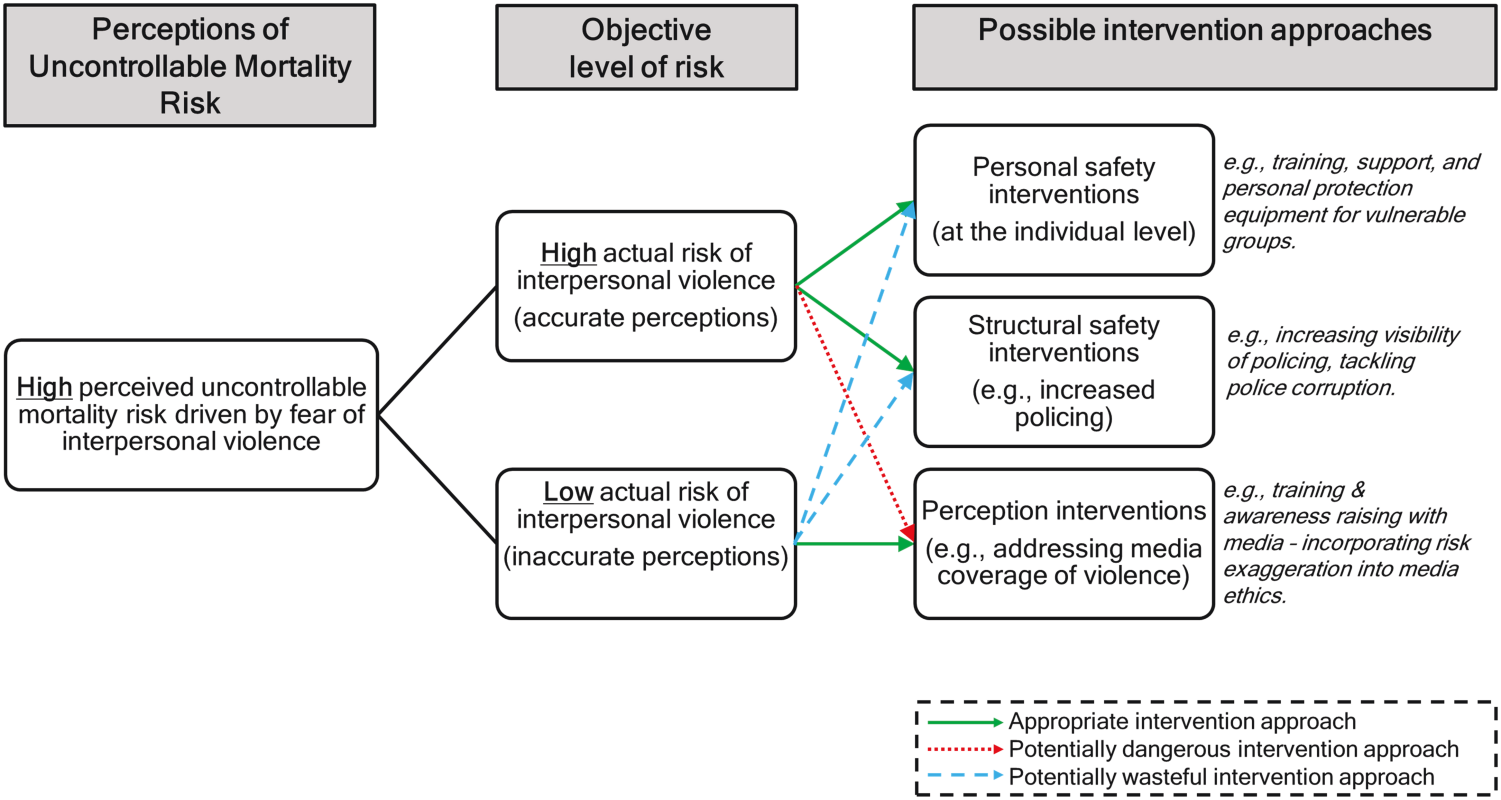
Infographic showing the benefit of comparing perceptions of uncontrollable mortality risk with objective measures of risk when assessing the suitability of interventions (using the example of the risk of interpersonal violence).

Finally, comparing perceptions of uncontrollable mortality risk with objective measures of risk exposure may also help to determine which intervention approaches are most appropriate. For example, in a situation where levels of perceived uncontrollable mortality risk are high, and driven by fears of interpersonal violence, it would be potentially dangerous to pursue interventions aimed at lowering the public’s sense of risk if their actual level of risk is high. This is because lowering perceptions of risk may lead to an increase in risky behaviours, in an already risky environment. Similarly, it would be a waste of resources to design structural interventions to tackle risk of violence in order to address elevated levels of perceived risk, in situations where the objective level of risk is low (see Fig. 3). As discussed, perceptions of uncontrollable mortality risk can influence preventative health behaviours. Assessing the accuracy of these perceptions may help to determine the suitability of different intervention approaches.

## Conclusion

The Uncontrollable Mortality Risk Hypothesis has a range of implications for future public health strategies. Structural changes to address environmental exposures to risk can lead to the double dividend of safety: the initial risk reduction, plus the subsequent secondary benefits from improved health behaviours. Public health strategies that consider the broader causal relationships between overall levels of uncontrollable mortality risk and multiple health behaviours, rather than focussing on narrow links between individual risks and obviously related behaviours, may enable more effective health interventions. By measuring perceptions of uncontrollable mortality risk, researchers can capture underappreciated health benefits of risk interventions. This may also help to determine the most effective and appropriate intervention approaches for addressing exposures to uncontrollable risk.

## Glossary of terms

1. *Adaptive behaviour*: refers to a behaviour of a living organism which, in the environment they inhabit, improves their chances of survival and ultimately of leaving descendants, in comparison with the chances of a similar organism that does not exhibit the specified behaviour [86].
2. *Proximate explanations*: of behaviour are concerned with the mechanisms that underpin a behaviour and how it works. They refer to accounts that point to a cause or event which is closest to, or immediately responsible for causing, an observed behaviour. These contrast with *ultimate explanations* of behaviour [16, 87].
3. *Ultimate explanations*: of behaviour are concerned with why a behaviour exists. They look to identify the underlying reason for how a behaviour has manifested through natural selection. Ultimate explanations are concerned with the *fitness* consequences and identify why it is (or is not) selected for by the process of natural selection. This contrasts with *proximate explanations* of behaviour [16, 87].
4. *Optimal*: strategies in the context of behavioural ecology are behavioural strategies that maximize *fitness*, given the relevant environmental circumstances [88].
5. *Fitness*: in the context of behavioural ecology typically refers to a measure of the contribution of an individual to the genetic composition of subsequent generations through its own offspring or the long-term growth rate of a lineage [89, 90].
